# Reproductive consequences of transient pathogen exposure across host genotypes and generations

**DOI:** 10.1002/ece3.8720

**Published:** 2022-03-21

**Authors:** María Ordovás‐Montañés, Gail M. Preston, Georgia C. Drew, Charlotte Rafaluk‐Mohr, Kayla C. King

**Affiliations:** ^1^ 54203 Department of Zoology University of Oxford Oxford UK; ^2^ 54203 Department of Plant Sciences University of Oxford Oxford UK; ^3^ Institute of Biology Freie Universitat Berlin Berlin Germany

**Keywords:** *Caenorhabditis elegans*, host–pathogen interaction, immunity, maternal infection, reproduction, transgenerational effects

## Abstract

To maximize fitness upon pathogenic infection, host organisms might reallocate energy and resources among life‐history traits, such as reproduction and defense. The fitness costs of infection can result from both immune upregulation and direct pathogen exploitation. The extent to which these costs, separately and together, vary by host genotype and across generations is unknown. We attempted to disentangle these costs by transiently exposing wild isolates and a lab‐domesticated strain of *Caenorhabditis elegans* nematodes to the pathogen *Staphylococcus aureus*, using exposure to heat‐killed pathogens to distinguish costs due to immune upregulation and pathogen exploitation. We found that host nematodes exhibit a short‐term delay in offspring production when exposed to live and heat‐killed pathogen, but their lifetime fecundity (total offspring produced) recovered to control levels. We also found genetic variation between host isolates for both cumulative offspring production and magnitude of fitness costs. We further investigated whether there were maternal pathogen exposure costs (or benefits) to offspring and revealed a positive correlation between the magnitude of the pathogen‐induced delay in the parent's first day of reproduction and the cost to offspring population growth. Our findings highlight the capacity for hosts to recover fecundity after transient exposure to a pathogen.

## Graphical abstract

Our findings highlight the capacity for wild nematode lineages to recover, in terms of their fitness, from transient pathogen exposure. In nature, nematodes are living in microbe‐rich habitats where they may routinely encounter pathogens. The ability to maintain their fitness after a brief insult would be advantageous.

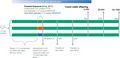

## 1 INTRODUCTION

Upon encountering a pathogen, hosts can pay costs from both direct pathogen exploitation and immune upregulation. These costs may occur even if a host is transiently exposed to a pathogen. During infection, hosts are hypothesized to invest more energy and resources to defense thereby reducing allocation to maintenance, growth, and reproduction, among other traits (Sheldon & Verhulst, 1996). Costs of immunity have been described theoretically and studied empirically in a diversity of host species (Schmid‐Hempel, 2013; Stearns, 1992), and studies have measured the negative effect of pathogens and/or immune stimulation on host metabolism (e.g., Bashir‐Tanoli & Tinsley, 2014), growth rate (e.g., Bartlett et al., 2018), and survival (e.g., Moret & Schmid‐Hempel, 2000). In terms of fecundity, infection has been found to drive both delays (Feistel et al., 2019) and reductions (Rigby & Jokela, 2000). In some cases, infected hosts reallocate resources to, or accelerate, reproductive output to offset higher host mortality (Forbes, 1993; Jokela et al., 1999). Further research is required to determine whether responses to transient pathogen exposure also vary across host genotypes, and whether the costs of these responses are carried across generations.

The consequences of pathogen exposure can affect host populations over generations (reviewed in Roth et al., 2018). Across host species, the parental, or even grandparental, experience of pathogen infection and/or immune challenge can generate a relative increase in offspring fitness, survival, and immune function (Ben‐Ami et al., 2020; Moret, 2006; Nystrand & Dowling, 2014; Tidbury et al., 2011). Less well established is the degree to which parental exposure and intergenerational immune priming carries costs for the recipient offspring (Contreras‐Garduño et al., 2014; Leponiemi et al., 2021; Zanchi et al., 2011). In some cases, costs become evident when the pathogenic environments of parents and offspring are mismatched (reviewed in Roth et al., 2018). These cases can occur when a pathogen is absent, in the next generation (Contreras‐Garduño et al., 2014; Nystrand et al., 2016), or when a different pathogen challenges the offspring (Sadd & Schmid‐Hempel, 2009). Another dimension to consider is whether these costs and benefits vary by host genotype. For intergenerational immune priming to evolve, it must be inherited and vary across host genotypes (Vu et al., 2015). Previous work in plants has shown that genotypes can differ in the type of mechanism utilized to pass information from parent to offspring (e.g., Castro et al., 2013; Galloway, 2001; Galloway & Etterson, 2007). The impact of parental infection on wild invertebrate populations remains to be fully determined in the context of multiple host genotypes. Host genotypes of a species can also differ in their ability to defend themselves against infection (e.g., Bartlett et al., 2018; Howick & Lazzaro, 2014; McKean et al., 2008) and might pay different costs of immune stimulation and/or pathogen exploitation (Valtonen et al., 2010). For example, host tolerance (Ayres & Schneider, 2012) and terminal investment (Duffield et al., 2017; Nystrand et al., 2016) are strategies which allow amelioration of some of the fitness costs from infection.

For many hosts, pathogen exposure may frequently be transient, and not the lifelong exposure simulated in many host–pathogen studies (e.g., Feistel et al., 2019; Petersen et al., 2015). For example, many host species are highly solitary and will have close interactions with conspecifics for short periods only, for example during mating. During these periods, transient exposure to new microbes is likely. Similarly, in wild populations, hosts may frequently be able to escape an infectious environment, via migration or changes to social behavior (Hurtado, 2008; Schmid‐Hempel, 2017; Shaw & Binning, 2020; Wilson & Sherman, 2010). Studies that have looked into varying exposure time found critical thresholds for when an infection overwhelms a host and recovery is no longer possible. Before these exposure thresholds, hosts transferred to pathogen‐free conditions can maintain lifespans found in control animals (e.g., Sifri et al., 2003; Tan et al., 1999). Transient exposure is ecologically relevant for many host systems, but it remains to be determined whether the costs stemming from brief exposure time vary across host genotypes.

*Caenorhabditis elegans* nematodes constantly interact with a species‐rich microbial community in their natural habitat of decomposing plant substrate (Félix & Braendle, 2010), including species of *Staphylococcus* (Montalvo‐Katz et al., 2013; Rossouw & Korsten, 2017). Dispersal to new locations on invertebrate vectors, and the dynamic nature of their habitat's microbial community (Schulenburg & Félix, 2017), means some of the nematodes’ exposures to pathogens may be transient in nature. This animal host is an established model for microbial pathogenesis (Gravato‐Nobre & Hodgkin, 2005) and immune response to pathogens (Pukkila‐Worley & Ausubel, 2012). In particular, *C. elegans* launches an immune response to both live and heat‐killed *S. aureus* after short‐term (8 h) exposure, but pathology is only observed in the live treatment (Irazoqui et al., 2010). *S. aureus*‐mediated killing is associated with the accumulation of live bacteria within the nematode gut (Sifri et al., 2003) where it produces toxins (Garsin et al., 2001). Within the first few hours of exposure, *S. aureus* colonizes the host gut and is able to persist past the termination of an 8h exposure (Irazoqui et al., 2010). *Caenorhabditis elegans* also shows evidence of infection‐induced maternal effects (Baugh & Day, 2020; Perez & Lehner, 2019), but the trade‐offs resulting from defense‐related multigenerational effects are not yet established (Willis et al., 2020). Wild *C. elegans* isolates demonstrate genetic variation in many phenotypes, including their response to pathogens, in terms of infection levels, pumping rate (a metric of pharyngeal behavior which indicates feeding), and evasion behavior (Schulenburg & Müller, 2004). Previous work on interactions between *C. elegans* and the pathogen *Bacillus thuringiensis* has also shown there is an evolutionary cost for maintaining immunity (Schulenburg & Müller, 2004).

The relative impacts of immune upregulation versus pathogen exploitation on host fitness, in total and over time, remain unclear (Schwenke et al., 2016; Sheldon & Verhulst, 1996). Here, we transiently exposed wild isolates and the lab‐domesticated isolate of *C. elegans* to live and heat‐killed pathogen, *S. aureus*. This approach allowed us to disentangle fecundity consequences stemming solely from an immune response, and those also driven by direct pathogen exploitation (Experiment 1). We then tracked the costs and benefits of transient pathogen exposure across successive host generations (Experiment 2). Exposures were followed by assays of host fecundity in the parental generation and in their subsequent three generations. This approach allowed us to test whether host genotypes that are suffering delays in terms of quantity (number of progeny) might have low‐quality offspring—if resources are lacking in infected parents—or high‐quality offspring—if parents were investing in priming.

## 2 MATERIALS AND METHODS

### 2.1 Nematode and bacterial strains

A diverse set of *C. elegans* isolates were selected from various geographical and genetic backgrounds (Table S1) that span the phylogenetic tree of Andersen et al. (2012). To represent wild hosts, we used *C. elegans* isolates CB4853, CB4854, CB4858, ED3017, JU1400, JU1490, JU258, LKC34, and QX1211 provided by the Woollard laboratory at the Department of Biochemistry, University of Oxford. To represent a lab‐domesticated host, we used the canonical wildtype strain N2 (Nicholas et al., 1959) that has been propagated in the lab for many generations and is genetically distinct from wild strains (Brenner, 1974, as detailed in Sterken et al., 2015).

Nematode populations were maintained at 20°C on Nematode Growth Medium (NGM; Brenner, 1974) with *Bacillus subtilis* PY79 food control (gifted by Lyle Simmons, University of Michigan) before being exposed to the pathogen *S. aureus* MSSA476. Given that *C. elegans* are bacterivores, and in the lab they derive their nutrition from monoaxenic bacterial lawns, we favored continuity between the food bacteria and pathogenic exposure. Both PY79 and *S. aureus* are gram‐positive and in the phylum Firmicutes. While *E. coli* OP50 has been historically chosen to maintain *C. elegans*, it is not a food source *C. elegans* would encounter in nature and is suboptimal in terms of development (Pang & Curran, 2014), metabolism (Brooks et al., 2009), and lifespan (MacNeil & Walhout, 2013). Maintenance of *C. elegans* on PY79 is not as widespread as that on OP50, but the former has been used as a control for *S. aureus* in previous studies (e.g., Garsin et al., 2001; Sifri et al., 2003), and PY79 does not upregulate the specific immune genes upregulated by live and heat‐killed *S. aureus* (Irazoqui et al., 2010). Heat‐killed pathogens, made and applied similarly, have been used in other studies as a no‐exploitation pathogen control for *C. elegans* (e.g., Morran et al., 2010 Science). Prior to exposures, batches of sterile age‐synchronized nematode eggs were prepared via bleaching (Stiernagle, 2006) and maintained at densities of ~1800 nematodes until L3/L4 stage.

The pathogen *S. aureus* was cultured at 30°C in Todd‐Hewitt Broth (3–5 ml) and the maintenance bacterium PY79 in LB (13–15 ml). For exposures, 55 mm Tryptic Soy Agar (TSA) plates were seeded with 60 μl of the MSSA476 or PY79 control at OD_630_ = 0.15. This concentration of liquid culture balanced the necessity to visualize nematodes while providing sufficient food. All maintenance and exposure plates were incubated at 30°C overnight. For exposures involving heat‐killed bacteria, overnight cultures were diluted to OD_630_ = 0.15 and incubated at 88°C for 1 h. Incubation conditions were determined by literature surveys and temperature trials (data not shown) to ensure no further bacterial growth occurred on plating. Heat‐killed samples were plated on TSA as described above.

### 2.2 Experiment 1: Effect of transient pathogen exposure on host fecundity and reproductive schedule

#### 2.2.1 Exposure of nematodes to bacterial pathogen

**FIGURE 1:**
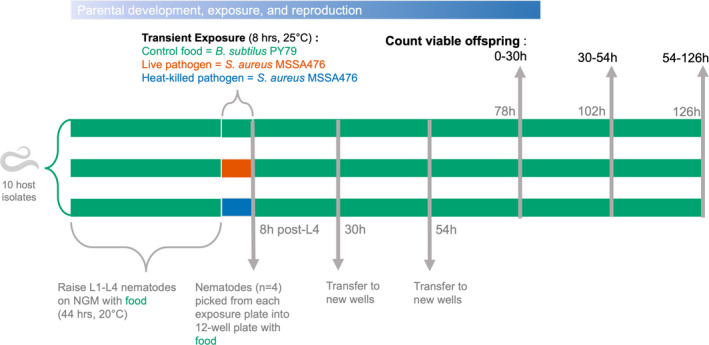
Procedure for *Experiment 1* testing the cost of pathogen exposure on cumulative progeny and lifetime fecundity of host nematodes. Experiments were performed across ten host isolates. Parental hosts developed on control food (green) for 2 days, before exposure to either control food, live pathogen (red), or heat‐killed pathogen (blue) for 8 h. Hosts were transferred back to control food for the remaining time. Transfers of parental nematodes occurred at 8 h, 30 h, and 54 h post L4 stage. Offspring from the first timeframe (eggs laid between 0 and 30 h) were counted 2 days later (approx. 78 h post L4), offspring from the second timeframe (eggs laid between 30 and 54 h) were counted 2 days later (approx. 102 h post L4), and offspring from the third timeframe (54–126 h) were counted on the final day. Blue gradient indicates progression in parental development. Time points during experimental course are denoted in gray font, timeframes at which eggs were laid are in black font

Approximately 100 nematodes (L3/L4 stage) were washed three times in M9 + Triton‐X and transferred to one of six replicate plates, with a lawn of either live or heat‐killed *S. aureus* or food control, and incubated at 25°C for 8 h (Figure 1). This exposure time was selected to maximize the period of immune upregulation while terminating before the host reproductive period. Previously N2 nematodes have been shown to express immune genes specific to *S. aureus* within this period, but have not yet started egg laying (Aprison & Ruvinsky, 2014; Irazoqui et al., 2010).

#### 2.2.2 Lifetime fecundity assays

To assess host fecundity after transient exposure to live and heat‐killed pathogen, nematodes (*n* = 4) were picked from each exposure plate (six replicate plates existed for each nematode isolate and exposure combination) into individual wells of a randomized 12‐well plate. Well plates contained 3 mL NGM agar and 30 μl of food. Nematodes were maintained at 25°C for the remainder of the experiment and were transferred to new wells with food each day, at 30 h‐ and 54 h‐post L4. Two days after each picking time point, live progeny from each single nematode were counted for 8–30 h, 30–54 h, and 54–126 h time intervals. Treatments consisted of six replicates. Overall, we tracked 720 founder nematodes over three time points. Nematodes that crawled off the plate or died from picking action at any time point were censored from the analyses (Table S2).

#### 2.2.3 Statistical analyses

All data were analyzed in R version 3.6.1 and RStudio version 1.2.1335 (R Development Core Team, 2019). All count data were checked for a normal distribution at each time point. The cumulative progeny from each founder nematode at each time point, for a given host isolate, and a specific bacterial exposure, were averaged for each replicate plate (Figure S1). These means were further averaged to combine the six replicate plates into one mean.

Comparisons of total brood size were analyzed using two‐way analysis of variance (ANOVA) with factors of host isolate and bacterial exposures, and also their interaction. This statistical approach was taken as we were unable to fit a generalized linear mixed‐effects model to the data due to a complex hierarchy in the experimental design and a logistically limited number of replicates. The number of offspring produced at 30 h (i.e., the first day of reproduction after pathogen exposure) was analyzed using a two‐way ANOVA to determine the presence of a reproductive delay among control and exposure treatments, across host isolates. The Tukey multiple comparison of means test was used to determine significant differences within host strains and bacterial exposures. Given normally distributed data, Pearson's product‐moment correlations were used to examine the relationship of mean brood sizes between nematodes exposed to control food and exposure treatments (Figure S2). It was evident that some hosts displayed consistently high reproduction (independent of the exposure); to account for this, we calculated ratios of relative fitness across the host isolates.

For each host isolate, relative fitness was calculated by dividing the number of progeny on pathogen exposure (either live or heat‐killed) over the progeny produced on control food (Figure S3). These relative fitness measures were calculated at the level of each biological replicate. Relative fitness data were analyzed at each time point with a binomial generalized linear model (GLM) with nematode isolate as an additional independent factor, followed by likelihood ratio tests and Tukey Contrasts for multiple comparisons of means with the car (Fox & Weisberg, 2019) and multcomp (Hothorn et al., 2015) R packages. Data were visualized using ggplot2 (Wickham, 2016) and RColorBrewer (Neuwirth, 2014) packages in R.

### 2.3 Experiment 2: Transgenerational impact of transient pathogen exposure

To assess the intergenerational impact of transient pathogen exposure, we tested selected host isolates (N2, CB4853, JU258, LKC34, and QX1211) based on the degree to which early fecundity was reduced by exposure in *Experiment 1*. We thus aimed to test whether host genotypes that suffer delays in producing progeny are more likely to have low‐quality or high‐quality offspring. Nematode populations were maintained at 20°C on Nematode Growth Medium (NGM) with *B*. *subtilis* PY79 (food control) before being exposed to *S. aureus* MSSA476 (pathogen).

#### 2.3.1 Transgenerational exposures to pathogen

**FIGURE 2:**
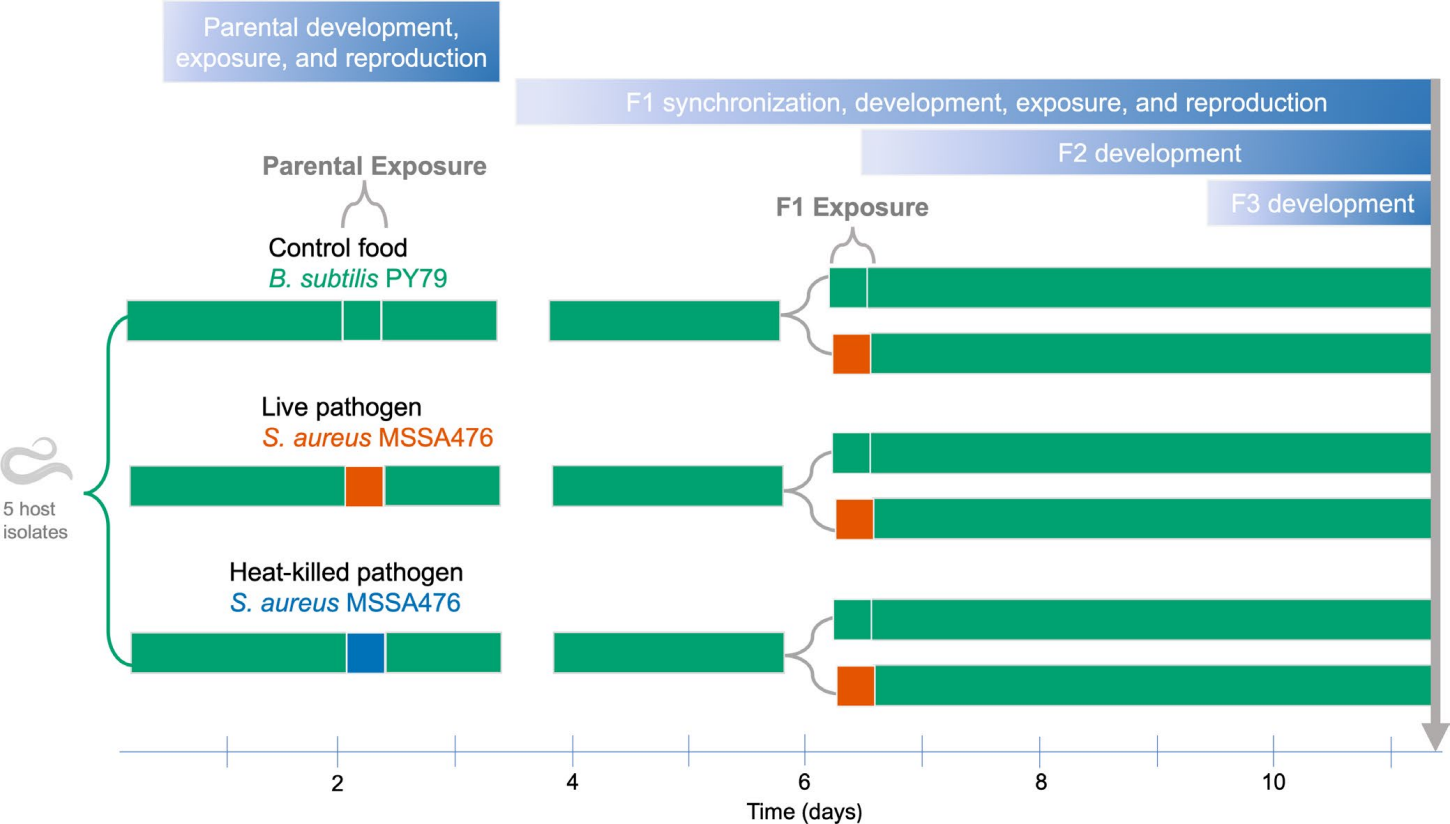
Design for *Experiment 2* which examines the pathogen exposure costs to offspring population expansion. Experiments were performed across five isolates. Parental nematodes developed on control food (green) for 2 days, before exposure to control food, live pathogen (red), or heat‐killed pathogen (blue) for 8 h. Nematodes were then allowed to reproduce for one subsequent day. The F1 generation was harvested and allowed to develop on food for 2 days. The 8 h exposures to food and live pathogen occurred for matched F1 nematodes derived from the same parental exposure plate. After exposure, single founder F1 nematodes expanded in population (F2 and F3) for 5 days. Blue gradients indicate progression in development. Downward gray arrow indicates final time point at which nematode population size was determined

The impact of repeated pathogen exposures in the parental (P) and offspring (F1) generations was evaluated by performing lineage expansion assays (see below) on host isolates (Figure 2). The P generation was synchronized via bleaching, then 1000 parental nematodes (L3/L4 stage) were exposed to food control or pathogen (live or heat‐killed) for 8 h at 25°C. Nematodes were then washed and moved to food for approximately 24 h to allow for egg production.

One day after pathogen exposure, the parents were bleached to yield the F1 generation, which was reared at densities of approximately 1800 nematodes per plate. The bleaching time matches the first time point in *Experiment 1* to allow the offspring from the “delayed reproduction” timeframe to be captured. This time point means that offspring had no direct contact with the bacterial exposure plate. After 44 h of development, matched L3/L4 stage F1 nematodes (*n* = 100 nematodes, derived from a common parental exposure plate) were exposed to either food control or live pathogen for 8 h. After exposure, three founder nematodes were picked off each exposure plate onto three separate 90 mm NGM plates seeded with 800 μl of food.

#### 2.3.2 Lineage expansion assays

To measure the impact of transient pathogen exposure in P and/or F1 generations on subsequent population growth, the number of individuals in a single host lineage across three generations (F1, F2, and F3) was counted. The method followed was similar to that described by Feistel et al. (2019). After 8 h exposure to either live pathogen or food control, three founder nematodes were picked off each exposure plate onto separate 90 mm NGM plates seeded with food. Plates were incubated at 25°C for 5 days while F1 nematodes produced F2 and F3 generations. On the final day, the average was taken from four 5 μl droplets and back‐calculated to give the nematode population size per plate. Treatments consisted of four biological replicates and three technical replicates. Population sizes were collected for a total of 356 founder nematodes (four nematodes were censored for leaving the plate or injury/death during transfer) (Table S4; Figure S4).

#### 2.3.3 Statistical analyses

Population sizes from three replicate nematodes from an exposure plate were combined to give a mean population size (Figure S5). We analyzed data from across host genotypes using a linear mixed‐effects model with host genotype, parental exposure, and F1 exposure (and their interactions) as fixed effects and batch as a random effect. Analyses were conducted using the lme4 (Bates et al., 2015) and car (Fox & Weisberg, 2019) packages in R. We conducted Tukey Contrast multiple comparisons of means tests with the multcomp package (Hothorn et al., 2015) to determine which host genotypes were significantly different from each other in terms of population size.

To examine the magnitude of both the benefits and costs of maternal effects, we calculated relative fitness ratios from the population sizes (Figure S6). A full breakdown of these calculations is shown in Table 1. To determine whether a correlation existed between benefits and costs of maternal effects, across host genotypes and each exposure condition, we used Spearman's rank correlation tests.

**TABLE 1:** Calculations for quantifying the relative costs and benefits of maternal pathogen exposure to host offspring

	Parental exposure	Offspring exposure	Calculation	Interpretation
Relative benefit				
*a*	HK	Live	x = $\frac{a}{\;c}$	If *x* > 1, then offspring exposed to live pathogen benefit from having a parent that was exposed to HK pathogen
*b*	Live	Live	x = $\frac{b}{\;c}$	If *x* > 1, then offspring exposed to live pathogen benefit from having a parent that was also exposed to live pathogen
*c*	Control	Live		
Relative cost				
*d*	HK	Control	x = $\frac{f}{\;d}$	If *x* > 1, offspring in a pathogen‐free environment carry a cost if they had a parent that was exposed to HK pathogen
*e*	Live	Control	x = $\frac{f}{\;e}$	If *x* > 1, offspring in a pathogen‐free environment carry a cost if they had a parent that was exposed to live pathogen
*f*	Control	Control		

We correlated the magnitude of the brood delay from the first day of reproduction after pathogen exposure (*Experiment 1*) with the cost to offspring (F1) population growth (*Experiment 2*). To calculate the magnitude of the brood delay, we took the cumulative progeny on the food control and divided it by the cumulative progeny after pathogen exposure; this was done using data from the 30h time point in *Experiment 1* (see Figure S3). This magnitude of delay was compared to the magnitude of the maternal effect cost using Spearman's rank correlation tests.

## 3 RESULTS

### 3.1 Experiment 1: Effect of transient pathogen exposure on host fecundity and reproductive schedule

**FIGURE 3:**
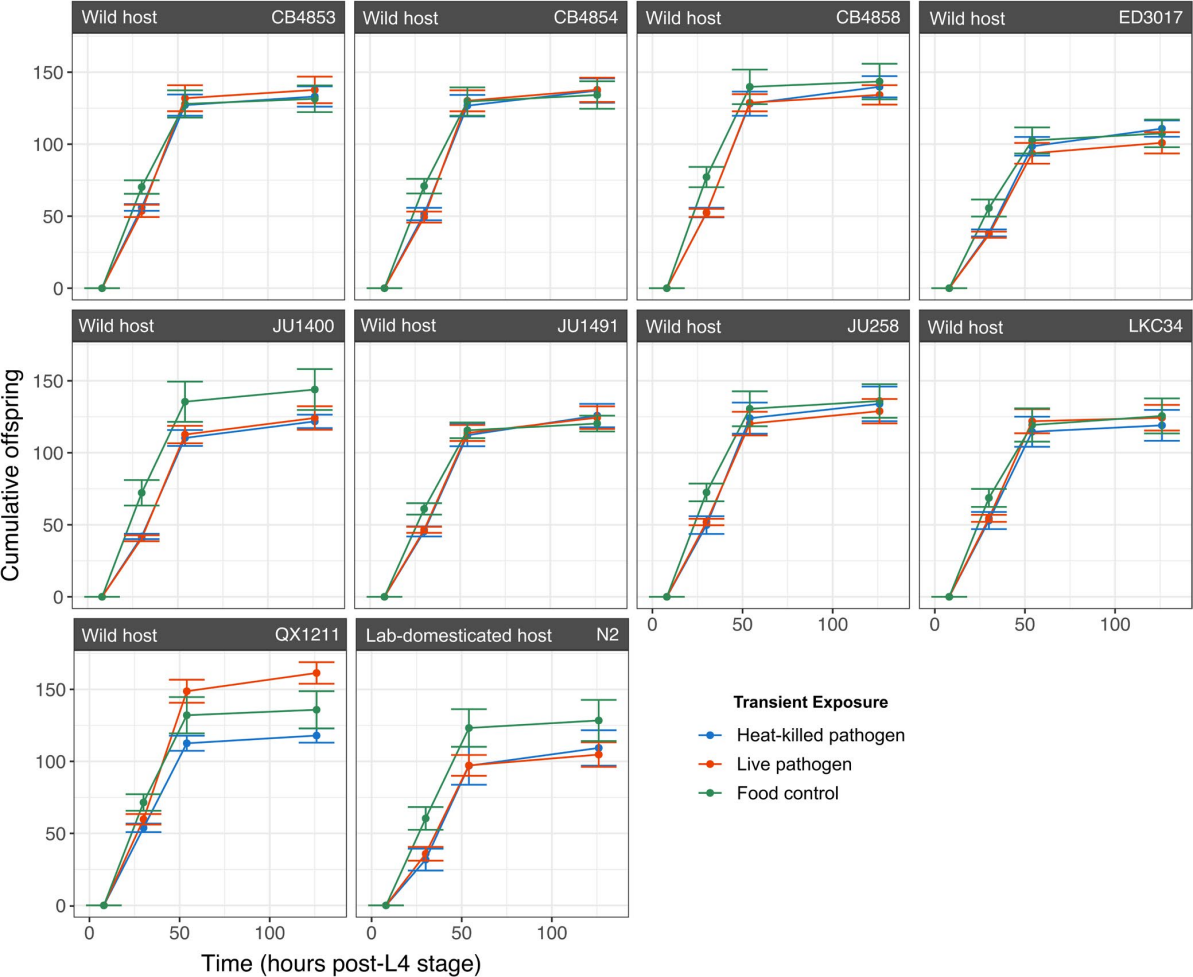
Cumulative viable offspring over time (mean ± 1 SE) for wild nematode isolates and lab‐domesticated N2 isolate. Nematodes were reared on food, then transiently exposed to live *Staphylococcus aureus* pathogen (red), heat‐killed *S. aureus* pathogen (blue), or *Bacillus subtilis* food control (green) in the first 8 h post L4 stage. All were moved onto food for their reproductive period. Host isolate origins and names are indicated above the grid

Here we sought to determine whether transient exposure to live and heat‐killed pathogen (*S. aureus* MSSA476) would reduce host lifetime fecundity, predicting that smaller broods would be attributed to both delays in reproduction and a general reduction in offspring. After 30 h post L4 stage, there were significant differences in cumulative offspring by bacterial exposure (Figure 3: ANOVA, *F* = 58.44, *df* = 2, *p* < .0001) and host isolate (ANOVA, *F* = 6.16, *df* = 9, *p* < .0001). There was no evidence for a significant interaction between exposure and host isolate (*F* = 0.54, *df* = 18, *p* = .93). Offspring counts at 30 h from hosts transiently exposed to live pathogen, and heat‐killed pathogen, were 33.4% and 34.5% lower, respectively, than those exposed to food (Tukey multiple comparisons of means, *p* < .001), pointing to a delay in reproduction. Despite this delay, exposed hosts recovered fecundity to control levels by the end of the reproductive period (Figure 3: ANOVA, *F* = 0.96, *df* = 2, *p* = .39). These results highlight that hosts transiently exposed to pathogens can suffer a delay in reproduction, but that lifetime fecundity can still recover to that of unexposed hosts.

**FIGURE 4:**
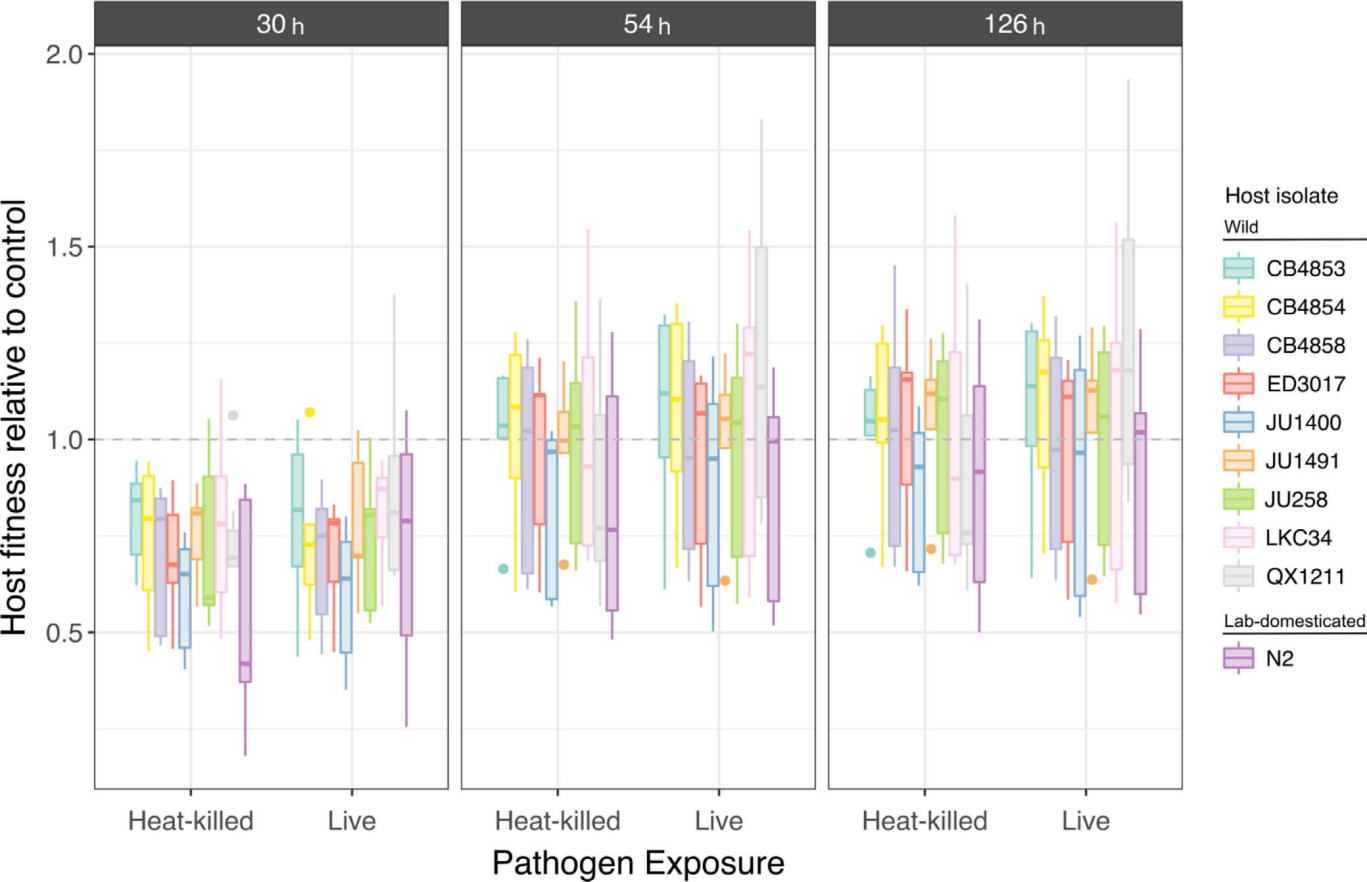
Relative fitness is expressed as host cumulative progeny on live or heat‐killed pathogen (*Staphylococcus aureus* MSSA476) relative to host cumulative progeny on food control. Relative progeny is shown from across the reproductive period at 30 h, 54 h, and 126 h post L4 and for all host isolates. Dotted line at *y* = 1 indicates when host reproduction after pathogen exposure is equal to host reproduction on food (i.e., comparable fitness). Relative to the controls, *y* > 1 denotes higher fitness and *y* < 1 denotes lower fitness. Boxes show first quartile, median, and third quartile of the data, whiskers extend 1.5 times the interquartile range from the first and third quartiles, and circles indicate outliers

Differences in cumulative offspring produced among host isolates were observed at 30 h (Figure 4: Binomial GLM, *χ* ^2^ = 44.79, *df* = 9, *p* < .0001), 54 h (Binomial GLM, *χ* ^2^ = 46.06, *df* = 9, *p* < .0001), and 126 h (Binomial GLM, *χ* ^2^ = 40.77, *df* = 9, *p* < .0001), irrespective of treatment. Notably, at 30 h, the lab‐domesticated host exhibited a greater fitness cost compared to wild isolates CB4853 (Tukey Contrasts, *p* = .0109), LKC34 (*p* = .0293), and QX1211 (*p* = .0495). There were no significant differences between pairs of hosts at subsequent time points. Across all time points, differences in pathogen treatments were not significant (Figure 4; Table S3). Thus, we did not detect an overall difference between the costs of immune upregulation and pathogen exploitation under our experimental conditions.

### 3.2 Experiment 2: Transgenerational impact of transient pathogen exposure

We next aimed to determine whether the pattern of delayed fecundity in *Experiment 1* could have lasting intergenerational impacts, for example, due to immune priming or other mechanisms. We created conditions where maternal stress matched offspring stress (matched pathogen exposure), and those where maternal stress was absent in the offspring's generation (mismatched pathogen exposure).

**FIGURE 5:**
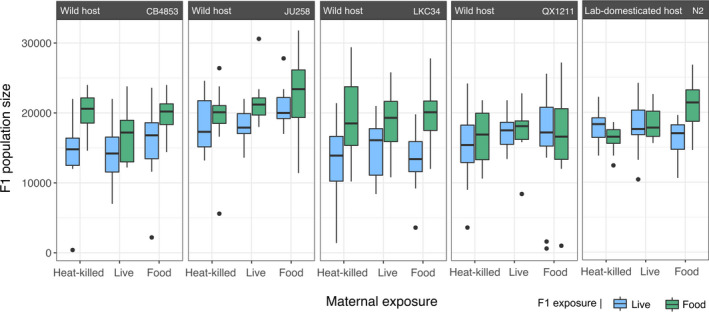
Population size across five host isolates after F1 nematodes were exposed to bacteria (live pathogen or food control) that matched or mismatched maternal exposures (heat‐killed pathogen, live pathogen, or food control). Boxplots display summarized data at the level of technical replicate (Figure S5), with each representing data aggregated from 12 lineage expansion plates (i.e., four batches each with three founder nematodes). The bacteria that maternal hosts were exposed to are indicated on the x‐axis, and the bacteria that the offspring (F1) were exposed to are represented by blue (live pathogen) or green boxplots (food control). Boxes show first quartile, median, and third quartile of the data, whiskers extend 1.5 times the interquartile range from the first and third quartiles, and circles indicate outliers

Within host genotypes, we found little evidence for maternal pathogen exposure having an intergenerational effect (Figure 5). Pathogen‐exposed (either live or heat‐killed) parents and unexposed parents all founded populations of a comparable size. This occurred despite the delayed reproduction reported in *Experiment 1*. Looking across host genotypes, we observed significant differences in population size (Figure 5: Linear mixed‐effects model, *χ* ^2^ = 34.13, *df* = 4, *p* < .0001). The LKC34 wild isolate had significantly lower population sizes compared to wild isolate JU258 (Tukey Contrasts, *p* = .0151) and lab‐domesticated N2 (Tukey Contrasts, *p* = .0349), independent of exposures in any generation. There was a significant effect of exposure (linear mixed‐effects model, *χ* ^2^ = 32.06, *df* = 1, *p* < .0001) with offspring producing lower population sizes on the live pathogen treatment compared to food (Tukey Contrasts, *p* = .0005). We also found an interaction between host genotype and F1 exposure (linear mixed‐effects model, *χ* ^2^ = 15.26, *df* = 4, *p* = .004). However, we found no interaction between the exposures of the P and F1 generations (linear mixed‐effects model, *χ* ^2^ = 1.54, *df* = 2, *p* = .46). We tested for the correlation between the magnitude of benefits of maternal pathogen exposure and the magnitude of such costs. We found no evidence of a correlation between the two metrics after either live (Figure S7a: Spearman's rank correlation, *p* = .78, rho = −.2) or heat‐killed (Figure S7b: Spearman's rank correlation, *p* = .35, rho = .6) maternal pathogen exposure.

**FIGURE 6:**
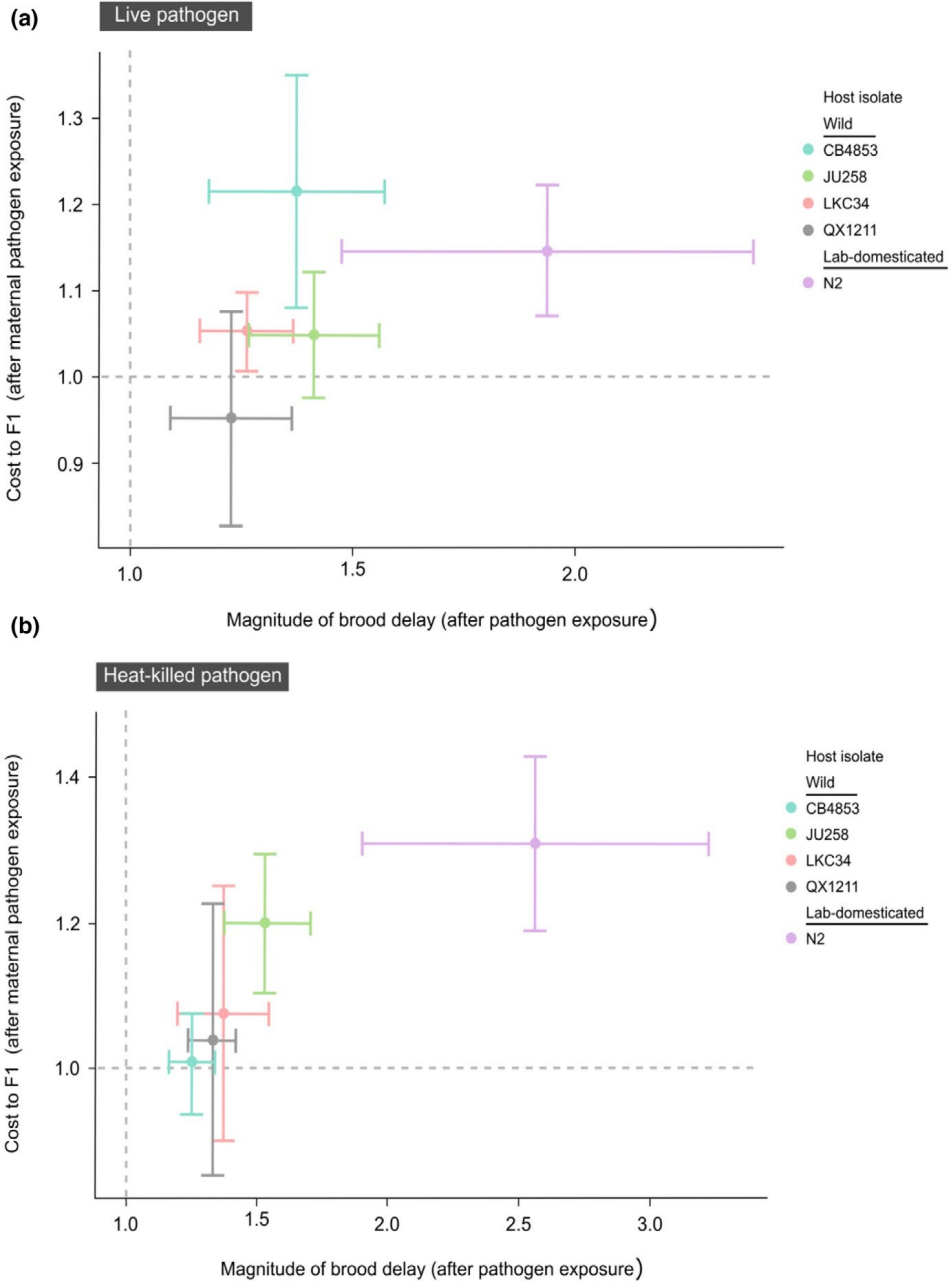
Magnitude of brood delay versus the cost to F1 of maternal exposure (mean ± 1 SE) for exposures to (a) live and (b) heat‐killed pathogen. Dotted line at *x* = 1 marks where cumulative offspring from parents exposed to pathogen equals the reproduction of hosts exposed to food control. Where *x* > 1, pathogen exposure is disadvantageous, with higher x values indicating poorer performance after pathogen exposure relative to food for a given host isolate. Dotted line at *y* = 1 marks where F1 exposed to food control expand their population equally well whether their parents were exposed to food control or pathogen (i.e., no cost of maternal pathogen exposure). If *y* > 1, it is disadvantageous for parents to have been exposed to pathogen, compared to parents on control food. If *y* < 1, it is advantageous. Note that more extreme delays in brood production and higher F1 population cost of maternal effects are both indicated as values >1

We also investigated potential trade‐offs in the magnitude of benefits and costs across one lab‐domesticated and four wild *C. elegans* isolates, and whether an association was evident between the delay in *Experiment 1* and the cost of maternal exposure in *Experiment 2*. We did not find a significant correlation for parents exposed to live pathogen (Figure 6a: Spearman's rank correlation, *p* = .45, rho = .5); however, a strong correlation existed across host isolates when parents were exposed to heat‐killed pathogen (Figure 6b: Spearman's rank correlation, *p* = .017, rho = 1). This result indicates that the reproductive costs of pathogen exposure can be mirrored in the next generation to some degree, even if this generation is sheltered from pathogens. Interestingly, some of the greatest reproductive costs across isolates were seen in the lab‐domesticated host, including the most severe delays in reproduction. The cost to the next generation also remained high for the lab‐domesticated host, but was particularly marked on exposure to the heat‐killed pathogen.

## 4 DISCUSSION

Host defense against pathogens is regarded as a costly life‐history trait that trades off with host reproduction (Stearns, 1992), but fitness costs of defense are not universally detected in host–pathogen interactions (e.g., Penley et al., 2018; Williams et al., 1999). We found that transient exposure to a pathogen caused a short‐term cost to host offspring production, but that over the host's lifetime, total offspring production was not adversely impacted. Hosts exposed to live or heat‐killed pathogen showed similar responses, suggesting that the small fitness costs that did manifest stemmed largely from immune upregulation rather than direct pathogen exploitation.

Some studies find costs manifest only in certain scenarios; this can depend on the density of competitors, nutrient availability, nutrient quality, and predation frequency (reviewed in Sandland & Minchella, 2003). For example, McKean et al. (2008) found costs to resistance and fecundity only in food‐limited environments. In the present study, hosts had access to food immediately after pathogen exposure; this switch to food may have facilitated clearance of pathogens from the gut (Sifri et al., 2003) and reduced the contribution of direct pathogen exploitation to the observed costs herein. We may only be able to detect the costs to lifetime fecundity and trade‐offs in a more stressful, food‐limited environment, or one in which there is constant exposure. Nevertheless, even though lifetime reproductive success across treatment was recorded as equal, the delay in reproduction represents a cost in populations where generations overlap and there is continuous population growth. In these cases, any lineages with delayed reproduction may ultimately have reduced representation at future time points.

We found a positive correlation between the magnitude of delay in reproduction and the cost experienced by uninfected offspring whose parents have been exposed to pathogen. Specific to host–pathogen interactions, immune priming between generations can prepare offspring for a future pathogen encounter (Roth et al., 2018), but can be costlier for populations if pathogen exposure reduces offspring fitness (e.g., Contreras‐Garduño et al., 2014; Sadd & Schmid‐Hempel, 2009). In other cases, the parental environment appears to have little effect on the offspring's fitness (e.g., Leung et al., 2013; Pansch et al., 2014). Immune challenged hosts frequently have lower quantity and/or quality offspring (reviewed in Schwenke et al., 2016), and transgenerational immune priming has been shown to incur fitness costs to the parent (Zanchi et al., 2012).

We did not find evidence of transgenerational immune priming against the pathogen *S. aureus*. In some cases, the induction of transgenerational immune priming can be dose dependent (Wilson et al., 2021; Wu et al., 2016). It is possible that transient exposures may not always allow pathogen densities to achieve required thresholds for induction.

We found that nematodes were largely resilient in overcoming the effects of their's, and their parent's, transient pathogen exposure. Resilience was especially high for wild isolates, while the lab‐domesticated host isolate tended toward producing lower‐quality offspring after pathogen challenge. Adaptations to a benign lab environment may carry consequences for host interactions with pathogens. When exposed to pathogens (which the lab lineage has not encountered in decades; Sterken et al., 2015; Weber et al., 2010), the lab‐domesticated hosts are not as prepared for defense compared to wild isolates, whose recent ancestors faced a multi‐microbial environment (Félix & Braendle, 2010). A caveat, however, is that only one lab‐domesticated isolate was used in this study. A more extensive test of the differences between domesticated and wild isolates may be possible in other systems, such as *Drosophila*, where numerous genetically distinct and lab‐adapted lineages are available (Faria & Sucena, 2017). Perhaps a stronger cost for all host isolates would be discernible under more naturalistic conditions. For example, these conditions could involve varying the duration of pathogen exposure or host developmental stage (e.g., Balla et al., 2015; Pereira et al., 2020), or by limiting food (e.g., Littlefair et al., 2017), or co‐exposing with other microbes of wild *C. elegans* (Félix & Braendle, 2010; Willis et al., 2020).

Transient exposure of wild nematode hosts to an opportunistic pathogen did not induce detectable lifetime fitness costs or costs to successive generations. Exposed hosts did exhibit short‐term reductions in offspring production, but were able to recover total fecundity once the pathogen source was removed and food was provided. Moreover, across wild host isolates and exposure conditions, pathogen‐exposed parents produce offspring that are of comparable quality to control offspring. For many host species, inducing a transgenerational response may not be worth the cost for transient exposures to pathogens which may easily clearly, or the host can move away from (Shaw & Binning, 2020). This interpretation may fit the lifestyle of these nematodes; *Caenorhabditis* constantly encounter microbes, ranging from mutualists to pathogens, in their environment that consists of decomposing substrates (Félix & Braendle, 2010). Offspring from pathogen challenged hosts in these environments may however experience a slight delay in their total fecundity; over time, this could have an adverse effect on their representation in the population.

## CONFLICT OF INTEREST

The authors declare no competing interests.

## AUTHOR CONTRIBUTIONS

**María Ordovás‐Montañés:** Conceptualization (equal); Data curation (lead); Formal analysis (lead); Funding acquisition (equal); Investigation (lead); Methodology (lead); Validation (lead); Visualization (lead); Writing – original draft (lead); Writing – review & editing (equal). **Gail Preston:** Conceptualization (equal); Data curation (supporting); Formal analysis (supporting); Investigation (supporting); Methodology (supporting); Project administration (supporting); Supervision (equal); Validation (supporting); Visualization (supporting); Writing – original draft (supporting); Writing – review & editing (supporting). **Georgia C Drew:** Data curation (supporting); Formal analysis (supporting); Validation (equal); Visualization (supporting); Writing – review & editing (supporting). **Charlotte Rafaluk‐Mohr:** Data curation (supporting); Formal analysis (supporting); Writing – review & editing (supporting). **Kayla King:** Conceptualization (lead); Formal analysis (supporting); Funding acquisition (equal); Investigation (supporting); Methodology (equal); Project administration (lead); Supervision (lead); Validation (equal); Visualization (supporting); Writing – original draft (lead); Writing – review & editing (lead).

## Supporting information

Supplementary Material

## Data Availability

The data underlying this study can be found at the figshare digital repository (10.6084/m9.figshare.c.5807957).
