# Supplementary material for: Reproductive consequences of transient pathogen exposure across host genotypes and generations
Source: Ecol Evol. 2022 Mar 21;12(3):e8720. doi: 10.1002/ece3.8720 (PMC8938310; doi:10.1002/ece3.8720)
Supplement: Supplementary file 1 — Supplementary Material [file ECE3-12-e8720-s001.docx]

**Supplementary Information**

**Reproductive consequences of transient pathogen exposure across host genotypes and generations**

María Ordovás-Montañés^1^, Gail M. Preston^2^, Georgia C. Drew^1^, Charlotte Rafaluk-Mohr^1,3^, and Kayla C. King^1^

^1^ Department of Zoology, University of Oxford, Oxford OX1 3SZ

^2^ Department of Plant Sciences, University of Oxford, Oxford OX1 3RB

^3^ Institute of Biology, Freie Universitat Berlin, 14195 Berlin

* Corresponding author: [kayla.king@zoo.ox.ac.uk](mailto:kayla.king@zoo.ox.ac.uk)

Supplementary Table 1: *C. elegans* wild isolates used in this project and source information. Isolate names represent “isotype sets” as described by Andersen *et al.* (2012). Locations, GPS coordinates, and dates are presented with the amount of detail provided by the original researcher. Comments as compiled by Andersen *et al*., where available. Adapted from Supplementary Table 1 in Andersen *et al*.

| Isolate | Location | GPS Latitude | GPS Longitude | Isolation Date | Sampled by |
| --- | --- | --- | --- | --- | --- |
| CB4853 | Altadena, USA | 34.189 | -118.131 | 1974/05 | C.D. Johnson |
| CB4854 | Altadena, USA | 34.189 | -118.131 | 1974/05 | C.D. Johnson |
| CB4858 | Pasadena, USA | NA | NA | 1973 | E.M. Hedgecock |
| ED3017 | Edinburgh, United Kingdom | 55.92 | -3.19 | 2004/12/03 | A. Cutter |
| JU258 | Ribeiro Frio, Madeira | 32.73 | -16.89 | 2001/10 | M-A. Félix |
| JU1400 | Sevilla, Spain | 37.3845 | -5.988 | 2008/03 | M-A. Félix |
| JU1491 | Le Blanc, France | 46.63 | 1.06 | 2008/08/17 | M-A. Félix |
| LKC34 | Unknown city, Madagascar | -18 | 46 | 2005/06/17 | V. Stowell |
| QX1211 | San Francisco, USA | 37.7502 | -122.4331 | 2007/11/26 | M. Rockman |

Supplementary Table 2: Number of observations for each host isolate and exposure combination at the final timepoint. Observations indicate technical replicates: non-censored nematodes with a maximum of 24 in treatments where no hosts were censored. All treatment combinations had a sample size of six biological replicate plates except ED3017 hosts exposed to heat-killed MSSA476 had five biological replicate plates. HK = heat-killed pathogen.

| Host isolate | Exposure | Observations |
| --- | --- | --- |
| CB4853 | MSSA476 HK | 21 |
|  | MSSA476 live | 23 |
|  | PY79 | 22 |
| CB4854 | MSSA476 HK | 22 |
|  | MSSA476 live | 19 |
|  | PY79 | 20 |
| CB4858 | MSSA476 HK | 20 |
|  | MSSA476 live | 21 |
|  | PY79 | 23 |
| ED3017 | MSSA476 HK | 17 |
|  | MSSA476 live | 21 |
|  | PY79 | 24 |
| JU1400 | MSSA476 HK | 20 |
|  | MSSA476 live | 24 |
|  | PY79 | 21 |
| JU1491 | MSSA476 HK | 21 |
|  | MSSA476 live | 23 |
|  | PY79 | 22 |
| JU258 | MSSA476 HK | 18 |
|  | MSSA476 live | 22 |
|  | PY79 | 22 |
| LKC34 | MSSA476 HK | 22 |
|  | MSSA476 live | 23 |
|  | PY79 | 19 |
| N2 | MSSA476 HK | 22 |
|  | MSSA476 live | 20 |
|  | PY79 | 22 |
| QX1211 | MSSA476 HK | 19 |
|  | MSSA476 live | 24 |
|  | PY79 | 21 |

Supplementary Table 3: Results from statistical tests by figure and time point (in hours). Metric indicates the type of data analyzed, with fitness costs representing progeny from *S. aureus* treatments relative to food. Exposure refers to the three bacterial treatments and Host indicates nematode isolate type. Exposure*Host denotes interaction effect.

| Figure | Time | Metric | Results |
| --- | --- | --- | --- |
| 3 | 30 | Cumulative progeny | Exposure: ANOVA, F = 58.44, df = 2,  *P* < 0.001  Host: ANOVA, F = 6.16, df = 9, *P* < 0.001  Exposure*Host: ANOVA, F = 0.54, df = 18,  *P* = 0.93 |
|  | 126 | Total brood | Exposure: ANOVA, F = 0.96, df = 2, *P* = 0.39  Host: ANOVA, F = 3.92, df = 9, *P* = 0.0002  Exposure*Host: ANOVA, F = 0.79, df = 18,  *P* = 0.487 |
| 4 | 30 | Fitness cost | Exposure: Binomial GLM*, χ*^2^ = 0.30, df = 1,  *P* = 0.584  Host: Binomial GLM*, χ*^2^ = 44.79, df = 9,  *P* < 0.0001  Exposure*Host: Binomial GLM*, χ*^2^ = 2.37,  df = 9, *P* = 0.984 |
|  | 54 | Fitness cost | Exposure: Binomial GLM*, χ*^2^ = 3.35, df = 1,  *P* = 0.067  Host: Binomial GLM*, χ*^2^ = 46.06, df = 9,  *P* < 0.0001  Exposure*Host: Binomial GLM*, χ*^2^ = 13.17, df = 9, *P* = 0.155 |
|  | 126 | Fitness cost | Exposure: Binomial GLM*, χ*^2^ = 1.28, df = 1,  *P* = 0.257  Host: Binomial GLM*, χ*^2^ = 40.77, df = 9,  *P* < 0.0001  Exposure*Host: Binomial GLM*, χ*^2^ = 21.56, df = 9, *P* = 0.01 |


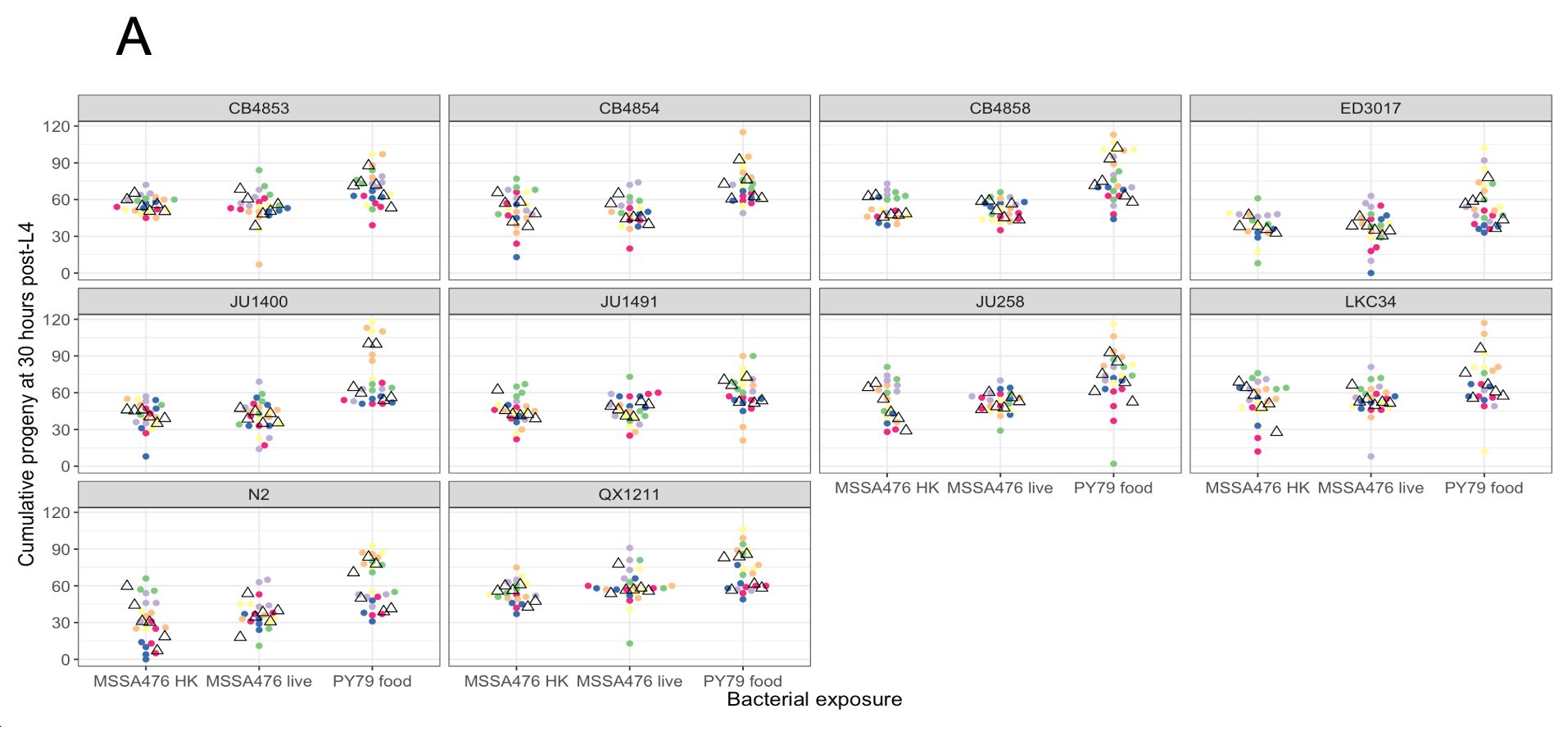


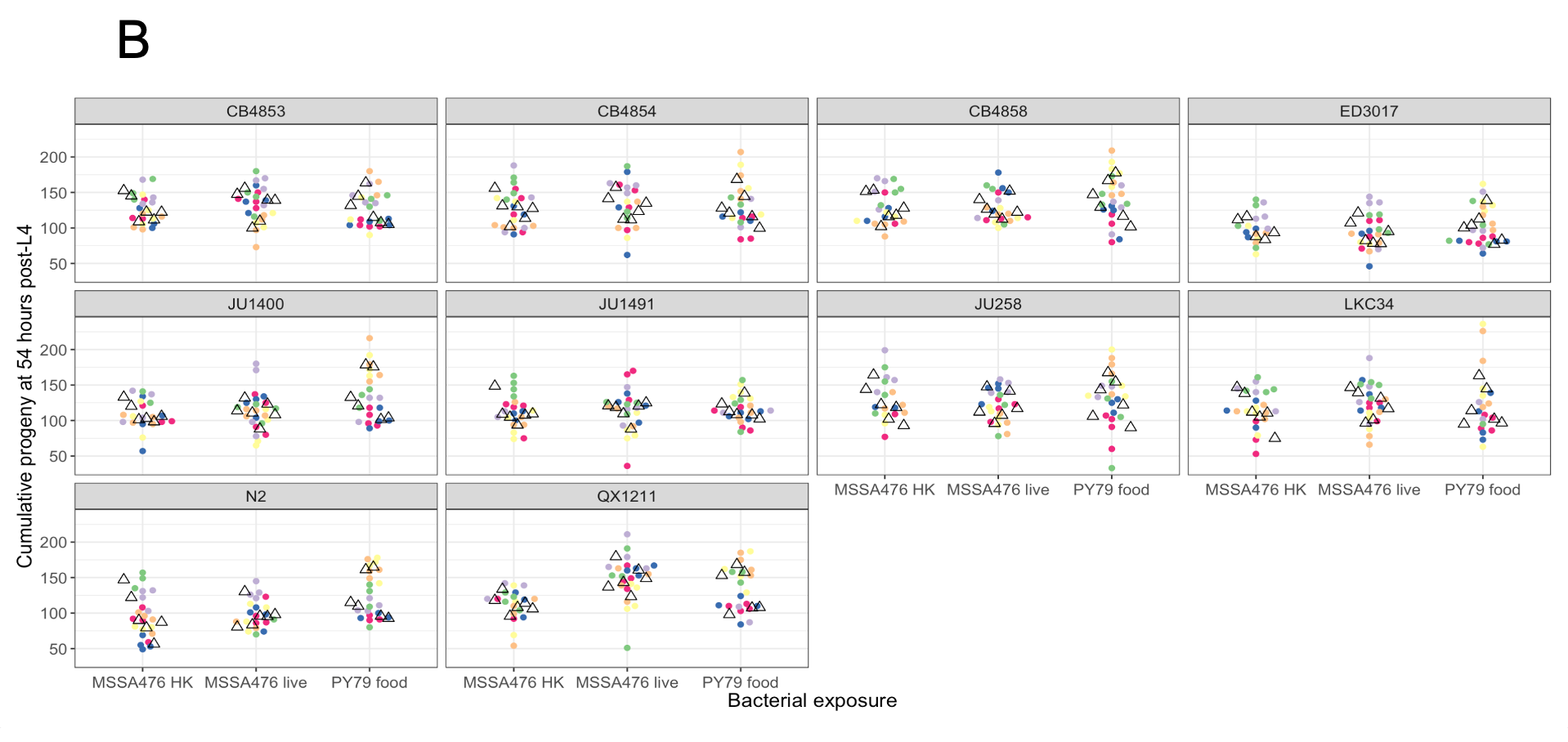


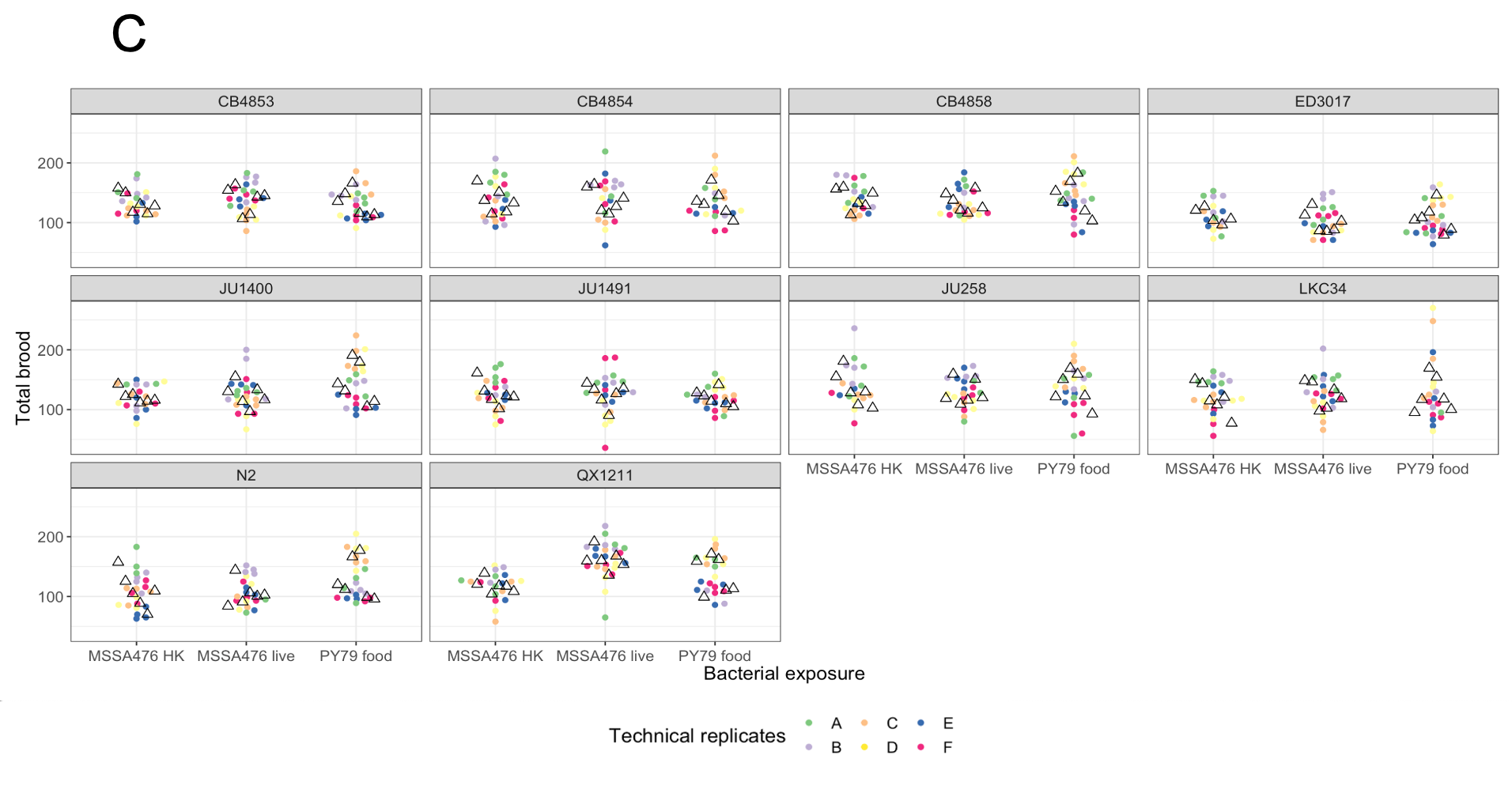


Supplementary Figure 1: Raw data showing variation in cumulative progeny of technical and biological replicates across (A) 30h, (B) 54h, and (C) 126h timepoints. Up to four technical replicates (founder nematodes tracked for reproduction over time) are depicted as points colored by their corresponding technical replicate code, A-F (A = green, B = purple, C = orange, D = yellow, E = blue, F = pink). The means of up to six biological replicates (exposure plates) are depicted as black triangles. Host isolate names indicated above each grid.


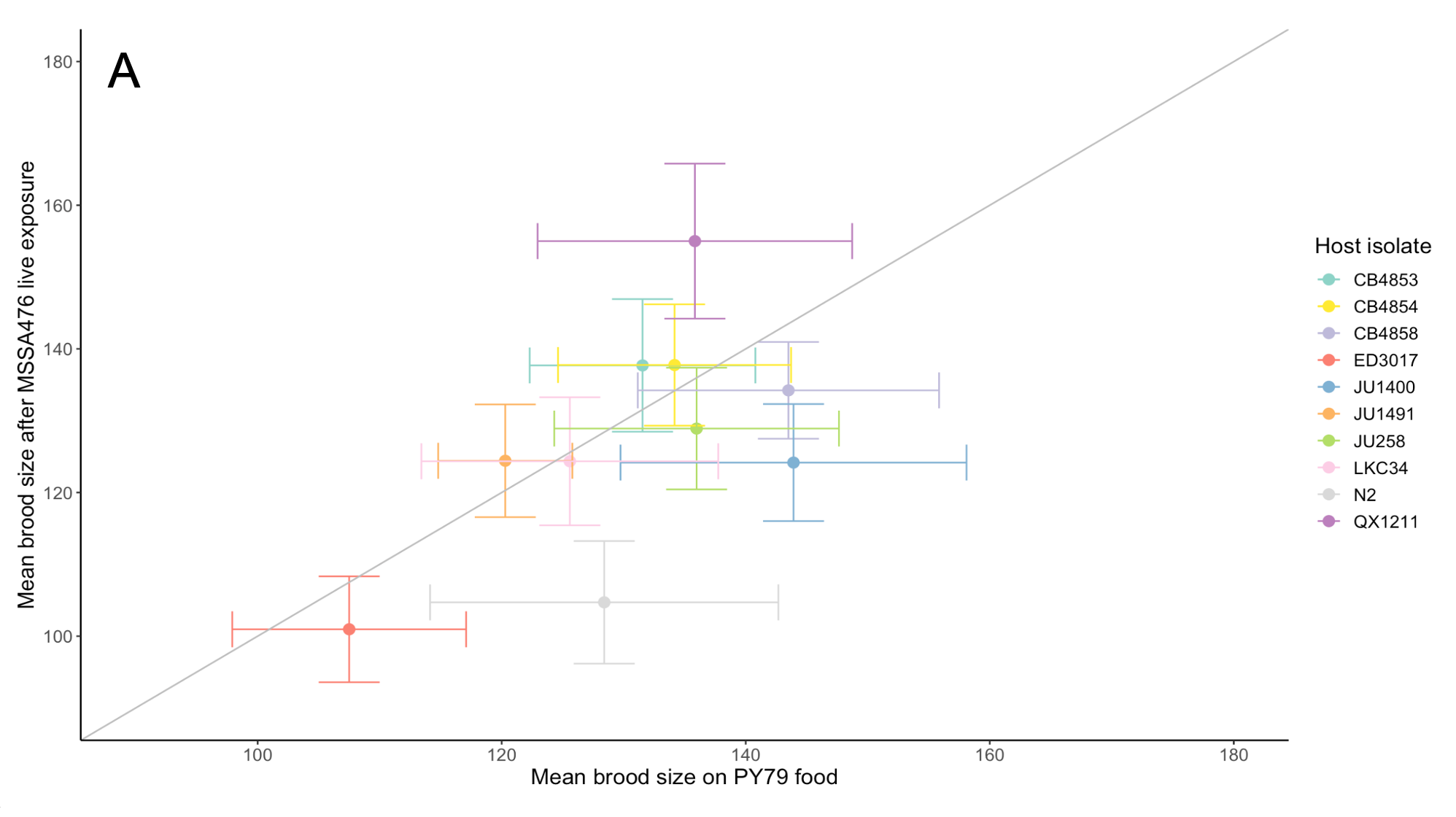

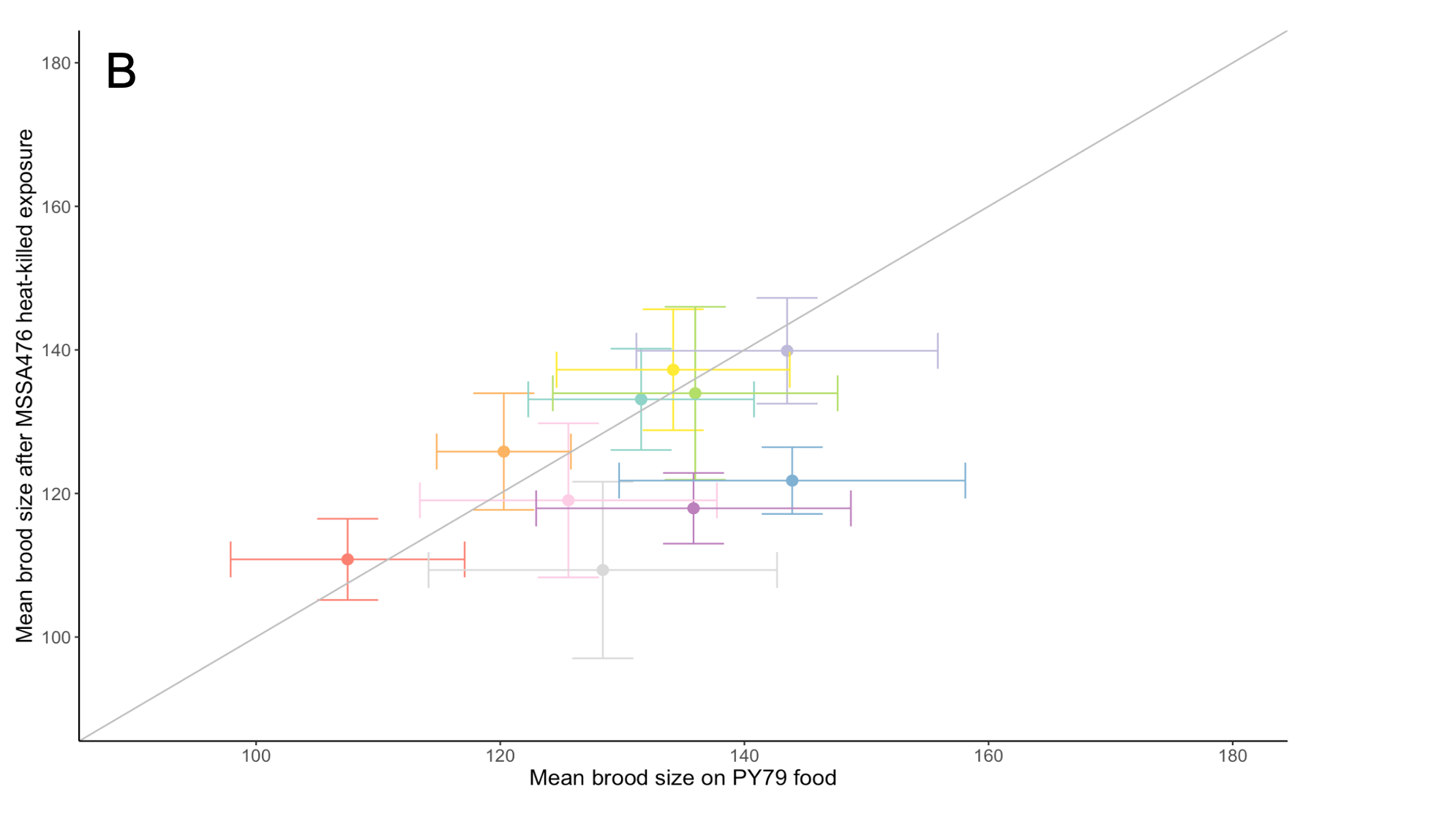


Supplementary Figure 2: Total number of offspring per nematode across ten isolates (mean ± 1 SE) on food and after exposure to either (A) live or (B) heat-killed *S. aureus* MSSA476. Grey line indicates y = x. Colors indicate ten host genotypes.


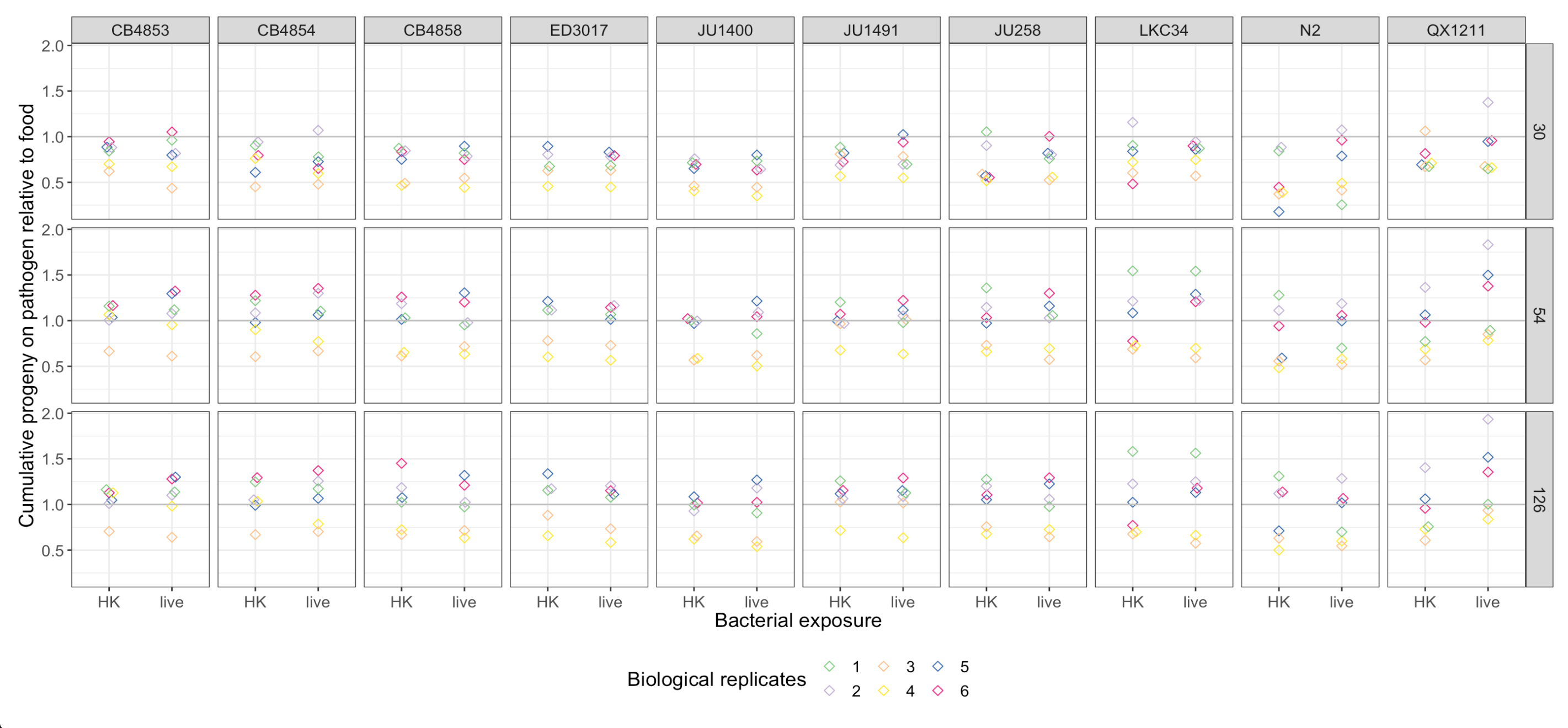


Supplementary Figure 3: Raw data showing the six ratios to represent fitness costs for a given bacterial exposure, host isolate, and timepoint. Ratios were constructed at the level of the biological replicate, by dividing cumulative progeny from hosts after MSSA476 heat-killed (HK on x axis) or MSSA467 live (live on x axis) treatments by the corresponding cumulative progeny on food with the same batch identity, 1-6. Host isolates are indicated along the top and timepoints (in hours) are indicated on the right. Grey line at y = 1 indicates when host reproduction after pathogen exposure equals host reproduction on food.

Supplementary Table 4: Number of observations for each host isolate, parental exposure, and F1 exposure combination. Observations indicate technical replicates: non-censored nematodes with a maximum of 12 in treatments where no hosts were censored. All treatment combinations had a sample size of four biological replicate plates. HK = heat-killed.

| Host isolate | Maternal exposure | F1 exposure | Observations |
| --- | --- | --- | --- |
| CB4853 | MSSA476 HK | MSSA476 live | 10 |
| JU258 |  |  | 12 |
| LKC34 |  |  | 12 |
| N2 |  |  | 12 |
| QX1211 |  |  | 12 |
| CB4853 | MSSA476 live |  | 12 |
| JU258 |  |  | 12 |
| LKC34 |  |  | 12 |
| N2 |  |  | 11 |
| QX1211 |  |  | 12 |
| CB4853 | PY79 |  | 12 |
| JU258 |  |  | 12 |
| LKC34 |  |  | 12 |
| N2 |  |  | 11 |
| QX1211 |  |  | 12 |
| CB4853 | MSSA476 HK | PY79 | 12 |
| JU258 |  |  | 12 |
| LKC34 |  |  | 12 |
| N2 |  |  | 12 |
| QX1211 |  |  | 12 |
| CB4853 | MSSA476 live |  | 12 |
| JU258 |  |  | 12 |
| LKC34 |  |  | 12 |
| N2 |  |  | 12 |
| QX1211 |  |  | 12 |
| CB4853 | PY79 |  | 12 |
| JU258 |  |  | 12 |
| LKC34 |  |  | 12 |
| N2 |  |  | 12 |
| QX1211 |  |  | 12 |


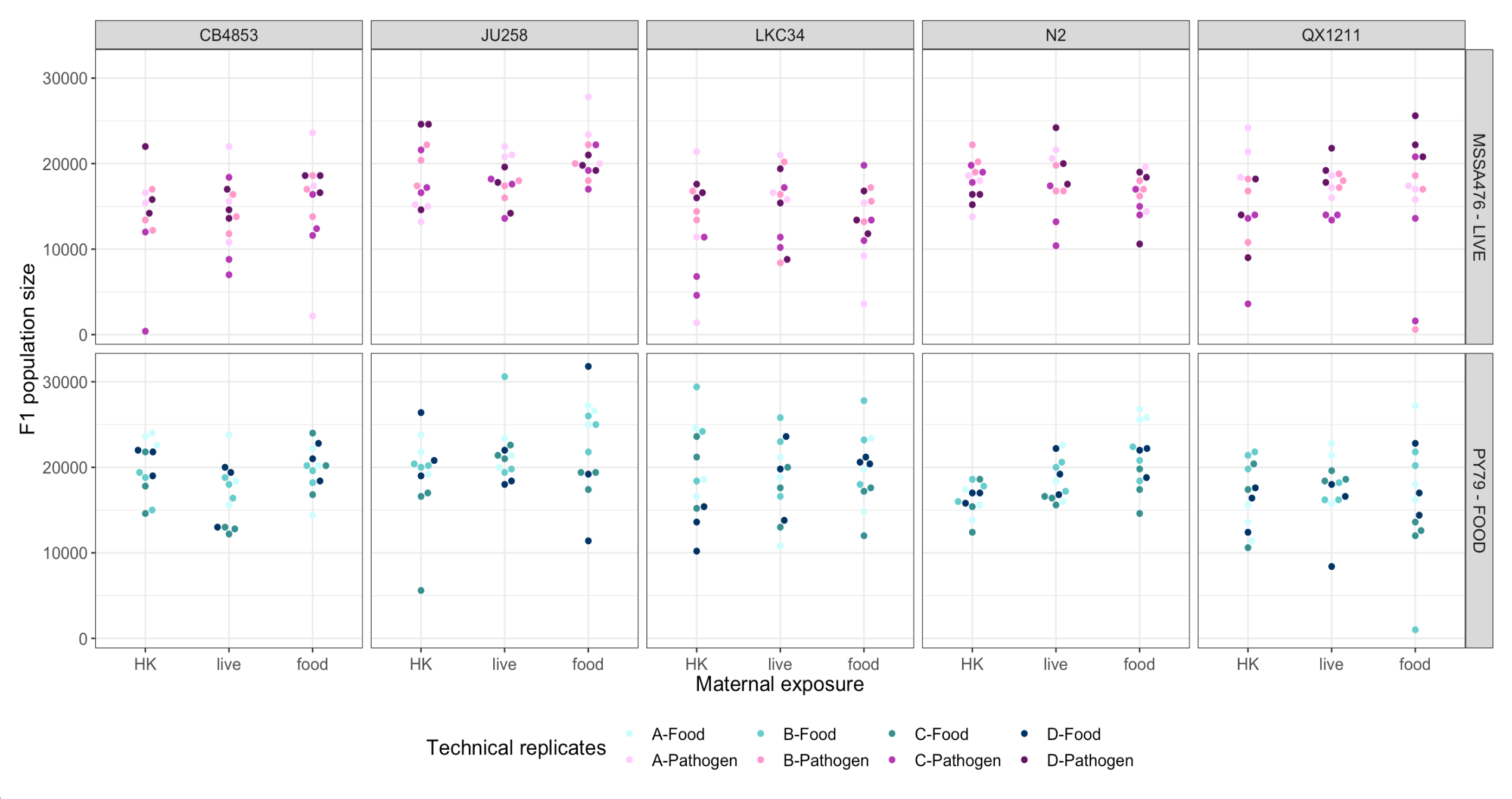


Supplementary Figure 4: Raw data showing variation in F1 population size of technical and biological replicates. Up to four technical replicates (founder nematodes tracked for population size) are depicted as points colored by their corresponding biological replicate code (A, B, C, or D). F1 pathogen exposure replicates are marked in the pink-purple gradient and control food replicates in the blue-green gradient. Nematode isolate names are shown on the top and F1 treatment is shown on the right. Maternal exposure is indicated on the x-axis as MSSA476 heat-killed (HK), MSSA476 live (live), and PY79 control (food).


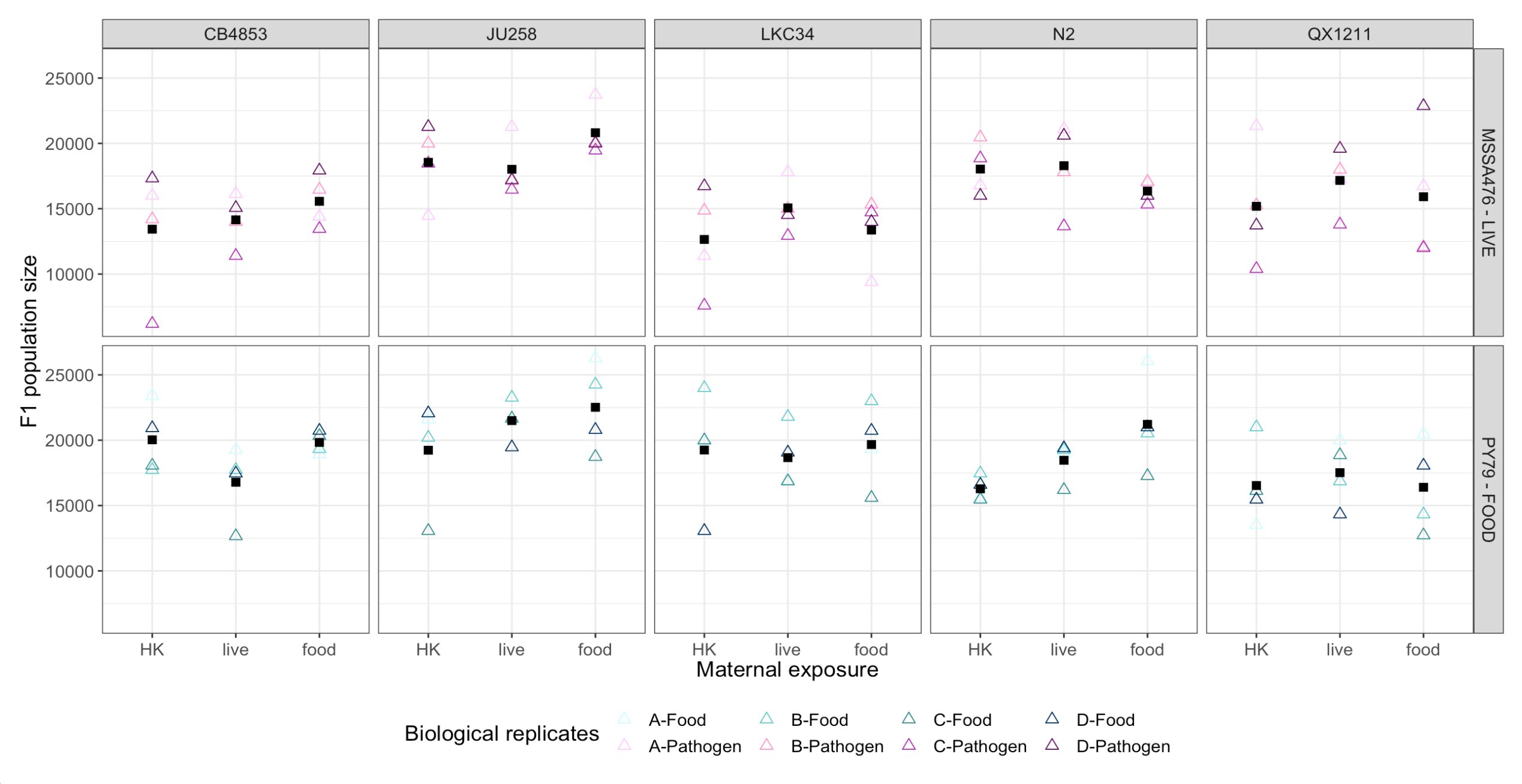


Supplementary Figure 5: Processed data showing the biological replicate averages of grouped technical replicates. The mean F1 population size of each biological replicate is shown as a triangle and the overall treatment mean is shown as a black square. Four biological replicates are shown (each colored for A, B, C, D) for each combination of worm isolate, maternal exposure treatment, and F1 exposure treatment. F1 pathogen exposure replicates are marked in the pink-purple gradient and control food replicates in the blue-green gradient. Nematode isolate names are shown on the top and F1 treatment is shown on the right. Maternal exposure is indicated on the x-axis as MSSA476 heat-killed (HK), MSSA476 live (live), and PY79 control (food).


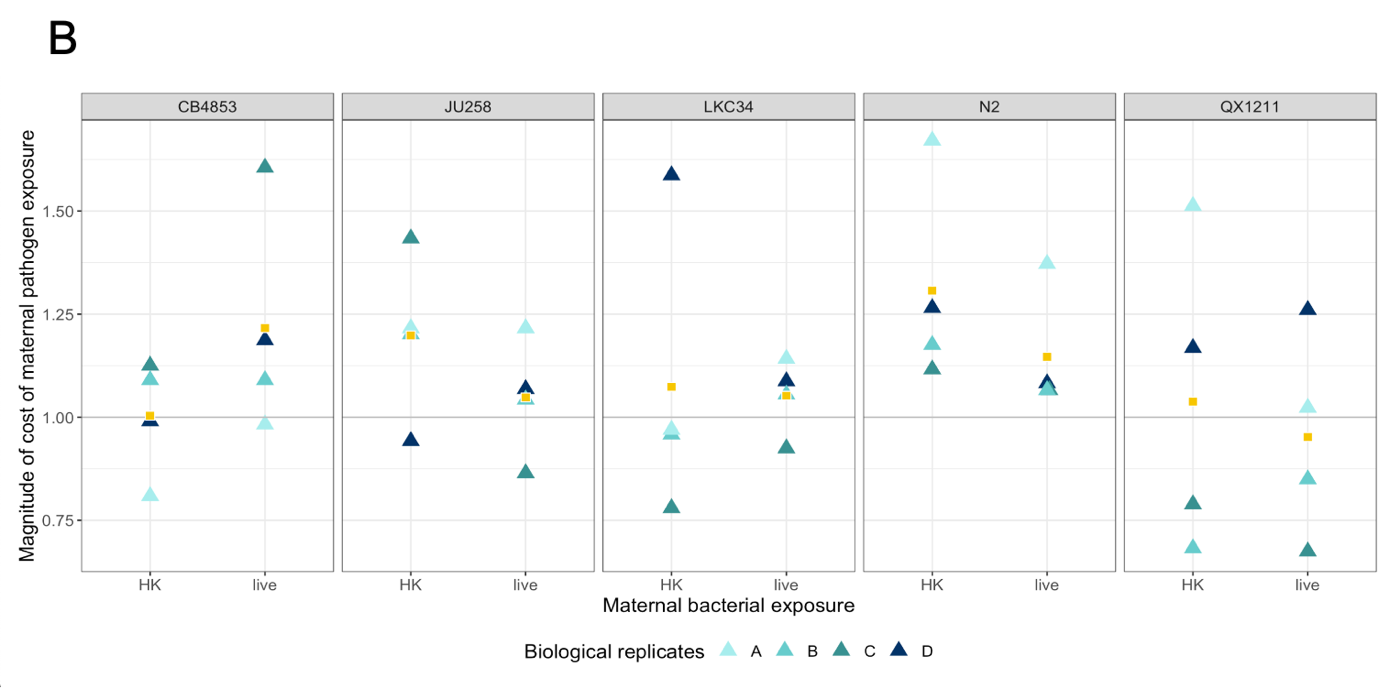

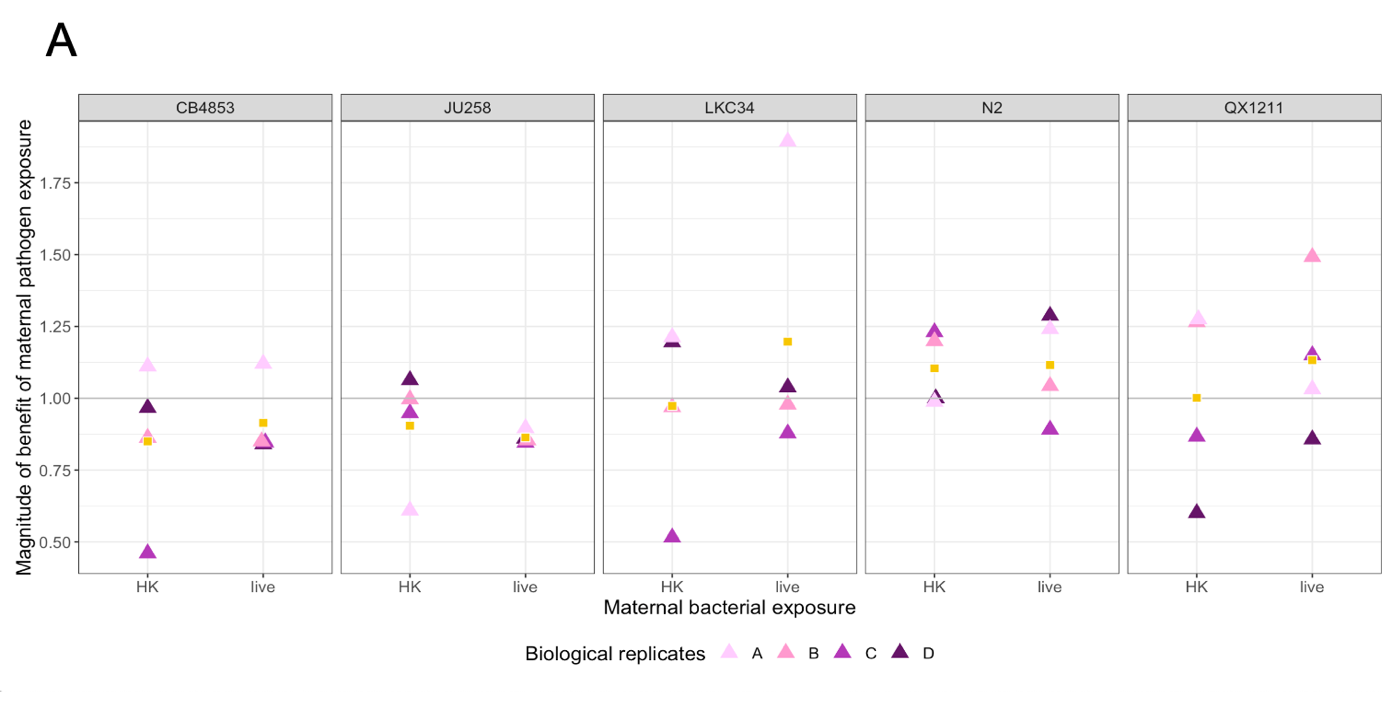


Supplementary Figure 6: Relative (A) benefit and (B) cost of maternal heat-killed and live pathogen exposure across nematode isolates. Triangles represent the magnitude of cost or benefit from maternal treatment and are colored by their biological replicate batch code. The benefit ratios represent the extent to which offspring whose mothers have been exposed to heat-killed or live pathogen reproduce more after live pathogen exposure (>1) than offspring whose mothers have continuously been on control food. The cost ratios represent the extent to which offspring whose mothers have been exposed to heat-killed or live pathogen reproduce less after food exposure and experience a greater cost (>1) than offspring whose mothers have continuously been on control food. The mean of each treatment is indicated with an orange square. They grey line at y = 1 represents the value where offspring encountering pathogen expand in population equally well whether their mothers have seen food or pathogen before (i.e. no benefit), and the value where offspring on control food expand in population equally well whether their mothers have seen food or pathogen before (i.e. no cost). Note that higher benefit and higher cost are both indicated as values >1. F1 pathogen exposure replicates (A) are marked in the pink-purple gradient and control food replicates (B) in the blue-green gradient. Nematode isolate names are shown on the top and maternal exposure is indicated on the x-axis as MSSA476 heat-killed (HK), MSSA476 live (live).


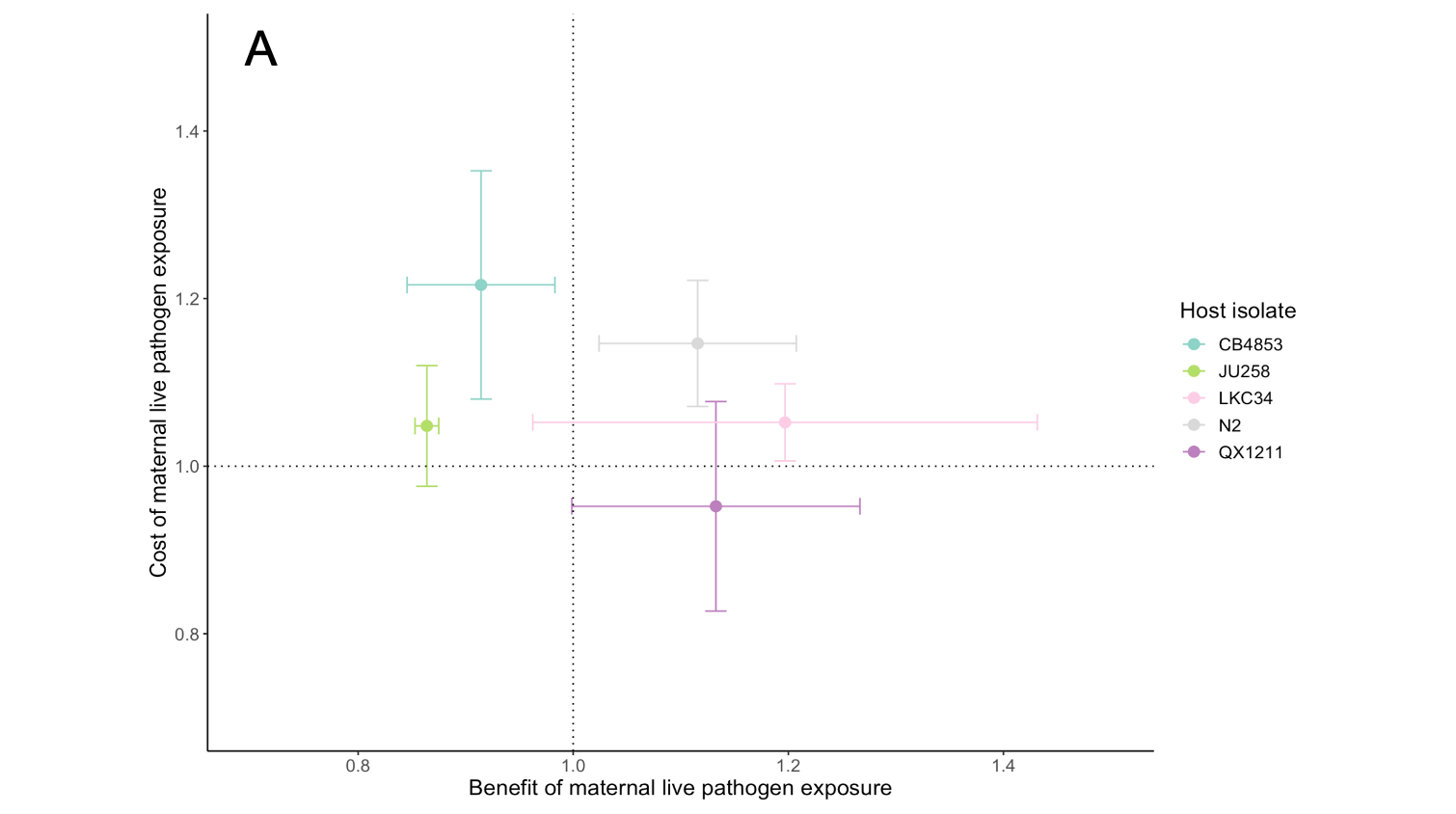

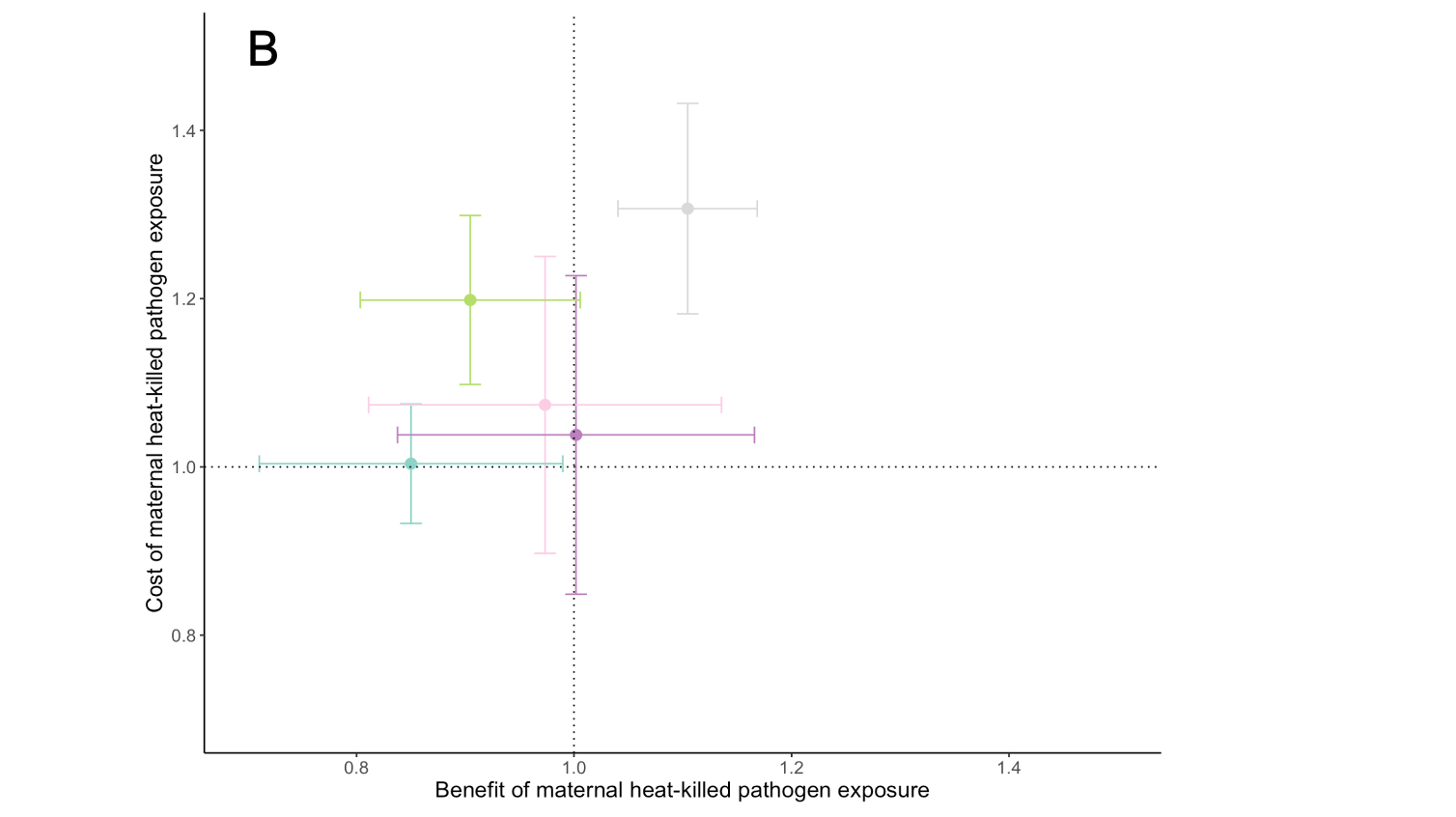


Supplementary Figure 7: Benefits versus costs of maternal (A) live and (B) heat-killed pathogen exposure on F1 lineage expansion. The relative benefits (x>1) are evidenced by the F1 live pathogen treatment and the relative costs (y>1) are evidenced by the F1 control food exposure (in both cases, comparing F1 from pathogen-challenged parents to F1 from control food parents). Dotted line at x = 1 represents threshold where F1 encountering pathogen expand their population equally well whether their parents have been exposed to food or pathogen (i.e. no benefit of maternal pathogen exposure). If x>1, it is advantageous for parents to have been exposed to pathogen, compared to parents on control food. If x<1, it is disadvantageous. Dotted line at y = 1 represents threshold where F1 encountering food expand their population equally well whether their parents have been exposed to food or pathogen (i.e. no cost of maternal pathogen exposure). If y>1 it is disadvantageous for parents to have been exposed to pathogen, compared to parents on control food. If y<1 it is advantageous. Note that higher benefit and higher cost are both indicated as values >1. Points and error bars represent mean ± 1 SE. Host isolates are indicated by colors in the legend.
